# Social costs associated with fibromyalgia in Spain

**DOI:** 10.1186/s13561-024-00527-1

**Published:** 2024-07-13

**Authors:** J Oliva-Moreno, C Vilaplana-Prieto

**Affiliations:** 1 Facultad de Ciencias Jurídicas y Sociales, Departamento de Análisis Económico y Finanzas, 45071 Toledo, Spain; 2 CIBER de Fragilidad y Envejecimiento Saludable, Instituto de Salud Carlos III, Madrid, Spain; 3 Department of Economic Analysis, University of Murcia, Murcia, Spain

**Keywords:** Fibromyalgia, Informal care, Labour productivity losses, Social costs

## Abstract

**Background:**

Fibromyalgia is a chronic rheumatic disease of unknown aetiology, highly disabling and mainly affecting women. The aim of our work is to estimate, on a national scale, the economic impact of this disease on the employment of patients and non-professional (informal) care dimension.

**Methods:**

Survey on Disabilities, Autonomy and Dependency carried out in Spain in 2020/21 was used to obtain information on disabled individuals with AD and their informal caregivers. Six estimation scenarios were defined as base case, depending on whether the maximum daily informal caregiving time was censored or not, and on the approach chosen for the valuation of informal caregiving time (contingent valuation and replacement time). Another six conservative scenarios were developed using the minimum wage for the estimation of labour losses.

**Results:**

Our estimates range from 2,443.6 (willingness to pay, censored informal care time) to 7,164.8 million euros (replacement cost, uncensored informal care time) (base year 2021). Multivariate analyses identified that the degree of dependency of the person suffering from fibromyalgia is the main explanatory variable for both the probability of being employed and the time spent in informal care. Conservative scenarios estimates range from 1,807 to 6,528 million euros.

**Conclusions:**

The high economic impact revealed should help to position a health problem that is relatively unknown in society and for which there are significant research and care gaps to be filled.

## Key Points

We estimate at national level (Spain) the economic impact of fibromyalgia on two dimensions: labour losses of patients and the value of informal care.

Our estimates range from 2,443.6 (more conservative scenario in the base case) to 7,164.8 million euros (less conservative scenario in the base case) (base year 2021).

The degree of dependency of the person suffering from fibromyalgia is the main explanatory variable for both the probability of being employed and the time spent in informal care.

## Background

Fibromyalgia is a highly disabling, chronic rheumatic disease of unknown aetiology, characterised by widespread musculoskeletal pain, which is accompanied by other symptoms such as fatigue, sleep problems, mental disorders and reduced functional capacity [1–6]. Prevalence figures estimated in different countries and studies vary widely, but the most common range is between 2 and 4% of the adult population affected, with young and middle-aged women being the population group where this prevalence is concentrated [7, 8].

People suffering from this disease are affected in various dimensions of their daily life, suffering physical and cognitive limitations, a low health-related quality of life and a significant impact on their productivity at work [9–20]. For instance, Espinoza et al. [20] estimated the consequences of pain in five different pathologies (knee osteoarthritis, hip osteoarthritis, lower back pain, shoulder pain, and fibromyalgia), measuring the losses in health state utilities for the affected population. The highest loss (in QALYs) corresponded to fibromyalgia followed by low back pain. The authors emphasized that these were long-standing pathologies, but also that patients faced a high component of psychological needs and multiple barriers both from the health system and social for their approach. In this sense, Salaffi et al. [19] showed that the probability of being employed ranged from 34 to 77%, with differences in social systems and labour markets being the underlying reasons for this wide dispersion. Other relevant effects involve family and social relations problems and challenges (difficulties with their partners, dependent on a family member in basic and instrumental activities of daily living, negative impact in relationships with children and friends …) [9, 10, 15, 21].

Although cost-of-illness studies in the field of fibromyalgia are not as abundant as in other pathologies, the scientific literature has revealed that the economic burden associated with this disease is heavy, regardless of the degree of severity of the disease, and that the total cost increases with the degree of severity [22–24]. Some studies also point that the burden of partner caregivers of fibromyalgia patients was positively related to greater functional disability and higher patient pain intensity [25]. The studies agree in identifying an intense use of healthcare services, and especially a high use of pharmaceuticals, primary care and rheumatology consultations. For instance, according to the study by Lacasse et al. [26] of patients diagnosed with fibromyalgia in Quebec (Canada), the highest direct costs corresponded to prescription drugs (some patients bought up to 13 different prescription drugs) and the second highest costs corresponded to consultations with health professionals other than physicians. Nevertheless, in studies that incorporate the social perspective, the costs deriving from the loss of productivity and other social costs (as informal care) far exceed the healthcare costs [22–24, 27–31].

To our knowledge, there are no studies that have attempted to estimate the economic burden of fibromyalgia at country level. The aim of our work was precisely this: to estimate the economic burden of fibromyalgia in Spain, restricted to the area of non-healthcare costs. More specifically, we estimated the cost of productivity losses of patients and the cost of non-professional or informal care associated with fibromyalgia in Spain in the year 2021.

Using the expression coined by Ghavidel-Parsa et al. [32], this paper proposes to uncover a part of the "iceberg-like" burden associated with fibromyalgia by stating the monetary value associated with the informal care received by people with fibromyalgia and the costs associated with not being able to continue working until retirement. In addition, our consideration of the degree of dependence and our application of various informal care valuation techniques allowed us to make comprehensive valuations and provide a realistic framework in which to estimate some of the costs of the hidden side of the underwater iceberg.

## Data and methods

The main source of data was the Survey on Disability, Personal Autonomy and Dependence Situations 2020 (EDAD2020). This was a macro-survey performed by the Spanish Statistics Institute (INE, in its Spanish acronym). The main objective was to "*meet the demand for information from the Public Administrations and numerous users such as organizations of the Third Sector of Social Action, providing a statistical basis for the planning of policies aimed at people with disabilities that allow the promotion of personal autonomy and the prevention of dependence situations*". It constitutes the only source of information about the health of caregivers of people with disabilities, as well as the time spent on caregiving and the consequences on their personal lives, both at work and in their leisure time.^1^

This survey was conducted in two phases: (i) a first phase (from August 2020 to January 2021) of localization of households in which people with disabilities and/or children with limitations resided, and (ii) a second phase (from April 2021 to October 2021) in which detailed information was collected about aspects related to disability (for people aged 6 and over), limitations (for children aged 2 to 5), services received and caregivers.

The survey was conducted throughout the Spanish national territory, using stratified sampling in two stages, with census sections and main family dwellings (110,130 dwellings) being the first and second stages. Stage 2 was designed to be conducted by CAPI (Computer-Assisted Personal Interviewing), but the pandemic situation made it difficult for an interviewer to visit the person directly, so the feasibility of CATI (Computer-Assisted Personal Interviewing) was explored. For this purpose, various tests were carried out, assessing whether the length of the telephone interview guaranteed the quality of the information, whether it kept the informant's attention until the end and whether it was necessary to adapt any questions to the new channel. The study showed that the CATI interview was feasible, and for this reason two collection methods were established: (i) CATI: consisting of computer-assisted personal interviewing, in which interviewers called households to conduct interviews by telephone. The informants could also call the toll-free number indicated in the letters previously sent to them announcing the survey, to request completion via this channel. (ii) CAPI: also consisting of computer-assisted personal interviewing, but in this case carried out by means of a personal visit by an agent with a portable device.

The survey collected information about: (i) the characteristics of the person with one or more disabilities (gender, age, nationality, marital status, completed studies, employment status), (ii) household equipment and conditions, net household income, disability domains (vision, hearing, communication, mobility, etc.), (iii) type of limitations (in children from 2 to 5 years old), (iv) health status and diagnosed diseases, (v) social and financial benefits received and (vi) formal and informal care received.

The EDAD2020 provides information about the officially recognized degree of dependence of those people who have already been assessed (see Appendix 1 for a description of the Spanish long-term care system). However, the interest was in determining the degree of dependence of everyone diagnosed with fibromyalgia, regardless of whether they had already been officially assessed or not. Given that the EDAD2020 Disability Questionnaire contains an extensive battery of questions (including questions about the degree of difficulty in performing activities of daily living without aids and without supervision, about the level of support required and about the impairment that has given rise to the disability), an adaptation of the EDAD2020 questions to the official assessment scale (Royal Decree 504/2007) has been carried out. To check the reliability of this procedure, the level of accredited dependence (for those people who have been officially assessed) was compared with the degree of dependence assigned using the EDAD2020. It was found that the degree of dependence using the EDAD2020 was higher than or equal to the accredited dependence, which is entirely plausible, since the dependence situation may have worsened since the official accreditation was received.

The EDAD2020 provides population weights corresponding to each individual, and which allow us to obtain population-level estimates. These population weights are provided through ratio estimators with a large sample size at the national level, which ensures unbiased estimates with little sampling error. Reweighting techniques (calibration) were applied according to sex, age and nationality, which allowed adjustment of the results of the deviations that occur due to the usual lack of response in some groups within the household surveys (for example, over-representation of elderly people).

### Labour productivity losses of patients

To estimate labour productivity losses, the theoretical framework applied was that of human capital [33–35], which considers that labour productivity can be reasonably approximated by the remuneration of labour factor, i.e. wages. Labour productivity losses were estimated in a two-stage process. First, the employment rates of fibromyalgia sufferers of working age (16–64 years) were compared with the employment rates of the general Spanish population, as provided by the Labour Force Survey of the National Institute of Statistics (Instituto Nacional de Estadística, INE). In this way, it was possible to compare the number of people affected by fibromyalgia who were actually working with the number of people expected to be working if they were not affected by fibromyalgia. Secondly, the estimated difference between these two figures was adjusted for the annual wages obtained from the Wage Structure Survey provided by the INE, adjusted by gender.

Although, in principle, it is to be expected that it is the presence of the disease that causes problems at work, it cannot be ruled out that people with a lower level of education are more exposed to the adverse effects of fibromyalgia and that this has a more intense and negative influence on their labour productivity. Therefore, we have carried out a conservative estimate (sensitive analysis) using the minimum interprofessional wage in force in Spain in 2021.

The EDAD2020 includes a question about whether the respondent has had to modify his/her working hours due to his/her disability or has had to change occupation or job within his/her company. Unfortunately, the questions are not asked in such a way as to allow us to value these changes monetarily.

It is important to note that the labour losses refer only to patients. In the case of carers, although their paid working time may be modified by the provision of their services, the valuation of their time is carried out using the techniques indicated in the following section.

### Assessment of informal caregiving time

Two approaches were used to assess informal caregiving time [36, 37]. First, in the replacement or ‘proxy good’ method, caregiving time was valued by taking into consideration the costs that would be incurred if informal caregiving did not exist, i.e., if informal caregiving were replaced by professional caregivers. To apply this method, the unit costs per hour of home-help service were used. The source of information was the official data from the IMSERSO/Ministry of Social Rights and Agenda 2030.^2^ The unit cost used was €15.66 per hour (base year 2021).

Secondly, contingent valuation (CV) techniques were used. These were the stated preference methods, indicating either willingness to pay (WTP) or willingness to accept (WTA). In our case, how much a person would be willing to pay for someone else to provide an hour of care in his or her place (WTP), or in exchange for what monetary amount a person would be willing to provide an additional hour of care (WTA). In this case, use was made of two recent Spanish papers [38, 39] that provide a range of values for WTP of €3.3—€5.6 per hour of care and for WTA of €6.4—€6.9. The original values of the items were updated to the year 2021, using the consumer price index.

The care time considered was the time reported by the main caregiver. In addition, a conservative criterion was used, limiting the maximum daily care time to 16 h [40, 41].

### Statistical methods

Two econometric analyses were carried out: for analysing the hours of informal care and the probability of being in work or being unemployed. In the first case, nonparametric robust regression was employed since it provides estimates that are robust to outliers and non-normality of residuals. It works iteratively by performing OLS regression to compute case weights based on absolute residuals and by re-running the regression using these weights until convergence. In the second case, probit models were estimated. Marginal effects were reported, as they could be interpreted as percentages, and the predicted probability of being in work, conditioned on the number of (censored or uncensored) informal caregiving hours, was also computed. As Hamilton [42] states, this method aims to achieve almost the efficiency of OLS with ideal data, and substantially better-than-OLS efficiency in non-ideal (e.g., errors are not normal, or not i.i.d.). Nonparametric robust regression starts by fitting ordinary least squares regression to identify Cook’s D value for each observation (i.e., measure the change in regression estimate if the observation is deleted). After highly influential outliers are set aside (observations with Cook’s Distance higher than 1), iteration process begins in which two types of weights are used: Huber weights [43] and biweights [44]. Both weighting functions are used because the former have problems dealing with severe outliers, whereas the latter sometimes fail to converge or have multiple solutions. Thus, the initial Huber weighting should improve the behavior of the biweight estimator. Standard errors are computed using the procedure proposed by Street et al. [45] for iteratively reweighted least squares.

All analyses were performed using the statistical software STATA 16.

## Results

According to the results obtained from the EDAD, the population with fibromyalgia in Spain and suffering from some type of disability amounted to 275,841 individuals, mainly female (89.7%), with an average age of 63 years. Low levels of completed education predominated (primary education and first stage of secondary education), with the most frequent marital status being married/partnered (47.9%), followed by widowed (20.5%). Only a small percentage of this population received formal/professional care, mainly in the form of home-help services. 57.0% of people with fibromyalgia received informal care, which means that at that population level the number of informal caregivers amounted to 157,086 people (approximately 77% of whom were co-resident caregivers). 70% were women, the average age was between 48 and 55 years, and the most frequent kinship relationships were son/daughter (49.0%) or spouse/partner (33.5%). The tasks mainly performed by caregivers are "shopping/preparing meals" (71.4%), "household chores" (63.4%), "going outside/walking" (48.5%) and "dressing/undressing" (47.1%).

Table 1 reflects the small percentage of the population with fibromyalgia and a disability who were in paid work while of working age. Only 12.5% of men and 21.9% of women were in paid employment. In comparison, 77.2% of men and 64.8% of women residing in Spain (general population) were in paid employment in 2021. Taking advantage of the larger sample size for women, we have introduced a breakdown into three age cohorts in Table 2. In this way, we can compare the evolution of the difference in the employment rate of women with fibromyalgia with respect to the total for Spain, according to age. Indeed, an inverted U-shaped behaviour can be observed. The difference between the two unemployment rates is 43 pp for women under 45 and those aged 55–64, but falls to 33 pp for the 45–54 age cohort.

**Table 1:** Description of people with fibromyalgia and informal caregivers. Population data

	Total	Care receiver’s sex	
		Men	Women
N (sample)	699	74	625
**At population level**	**275,841**	**28,471**	**247,370**
**Care receiver**			
Man	10.59	100.00	-
Women	89.41	-	100.00
Age	63.41	66.92	63.00
	(13.30)	(13.11)	(13.27)
Level of education			
Without elementary education	24.21	26.55	23.94
Elementary	21.29	24.91	20.88
Secondary	40.76	33.89	41.54
Tertiary	13.33	14.65	13.17
Missing	0.41	0.00	0.47
Marital status			
Single	12.57	14.94	12.29
Married/cohabiting	47.91	64.95	45.95
Widowed	20.48	9.55	21.73
Legally separated	5.02	2.9	5.27
Divorced	14.02	7.66	14.76
Dependency degree			
Non eligible	69.5	69.22	69.53
Moderate	19.31	19.76	19.25
Severe	6.03	4.2	6.24
High	5.17	6.82	4.98
Relation with economic activity			
Working	11.73	4.05	12.64
Unable to work	74.68	93.24	72.48
Unemployed	13.02	2.70	14.24
Missing	0.57	0.00	0.64
Receives from SAAD			
Cash subsidy	3.17	1.56	3.36
Home care	9.26	2.49	10.03
Day centre	2.39	0.00	2.67
Household employee	5.24	4.83	5.29
Receives care from informal caregiver	56.95	54.54	57.07
Co-resident caregiver	77.03	85.75	76.27
Non coresident caregiver	22.97	14.25	23.73
**Informal caregiver**			
At population level			
Total	**157,086**	**15,529**	**141,182**
Co-resident informal caregivers	121,003	13,315	107,688
Non-coresident informal caregivers	36,008	2,214	33,494
Man	30.65	22.39	31.64
Woman	69.35	77.61	68.36
Age	54.61	48.81	55.37
	(10.52)	(13.86)	(10.73)
Kinship with respect to care receiver			
Spouse/partner	33.45	50.00	31.30
Mother/father	3.04	5.88	2.67
Daughter/son	48.99	41.18	50.00
Other relative	14.53	2.94	16.03
Task primarily undertaken when providing care:			
Eating, drinking, feeding	10.60	9.85	10.68
Dressing / undressing / fastening of shoes	47.14	61.01	45.67
Grooming / dressing	51.89	71.72	49.79
Going to the toilet, changing diapers, etc.,	11.91	19.47	11.11
Changing posture/moving/keeping the body in a certain posture	21.99	47.58	19.27
Walking or moving around the house	19.32	33.32	17.84
Going outside, going up or down stairs, walking	48.53	30.76	50.41
Taking medication, going to doctor's appointments	40.87	53.63	39.52
Shopping, preparing meals	71.37	35.36	75.18
Carrying out other household chores	63.43	43.24	65.57
Using the telephone, computer, tablet, social networks	2.60	6.03	2.24
**Size of municipality**			
More than 100,000 inhabitants	43.78	41.89	44.00
50,000–100,000 inhabitants	12.73	9.46	13.12
20,000–50,000 inhabitants	12.73	10.81	12.96
10,000–20,000 inhabitants	13.16	16.22	12.80
Less than 10,000 inhabitants	17.60	21.62	17.12

Source: Own work using EDAD2020

**Table 2:** Labour participation. Comparison between population with fibromyalgia and general population

	Working	Total	Employment rate (%)	Employment rate for Spain (%)
Patient with fibromyalgia				
Men	1,321	10,576	12.49	77.21
Women	31,067	141,630	21.94	64.77
Less 45 years	7,333	22,070	33.23	72.64
45–54	12,145	47,985	25.31	68.27
55–64	11,588	71,576	16.19	49.30

Source: own work using EDAD2020 and Labor Force Survey. INEbase / Mercado laboral /Actividad, ocupación y paro /Encuesta de población activa / Últimos datos

The employment rate is the percentage of employed persons in relation to the comparable total population

The estimated monetary loss for reduced working associated with fibromyalgia was 1,438 million euros (Table 3). Of the total estimated loss, 132 million euros was for men (9.2%) and 1,306 million euros for women (the remaining 91.8%).

**Table 3:** Labour losses due to reduced labour participation associated to fibromyalgia in Spain (2021)

	**Patient: Men**		**Patient: Women**		**Total**	
	N (persons)	Wages foregone (million €)	N (persons)	Wages foregone (million €)	N (persons)	Wages foregone (million €)
Patient with fibromyalgia						
Average wage (by sex)	4,623	132.0	54,674	1,306.0	59,296	1.438.0
Minimum wage	4,623	62.5	54,674	738.6	59,297	801.1

Source: own work using EDAD2020, Labor Force Survey. INEbase / Mercado laboral /Actividad, ocupación y paro /Encuesta de población activa / Últimos datos and average wage by sex from Wage Structure Survey (INEbase / Mercado laboral /Salarios y costes laborales /Encuestas de estructura salarial / Últimos datos

Table 7 in Appendix 2 shows the results of the estimates of probit models for the probability of being employed and the probability of being unemployed. In the first case, taking the entire working-age population as a reference, a binary variable was defined that took the value 1 if the individual was employed, and 0 otherwise. In the second case, taking the active population (employed and unemployed, but not inactive people) as a reference, a binary variable was defined that took the value 1 if the individual was unemployed, and 0 otherwise. The explanatory variables were introduced progressively. In model M1, only age, sex and degree of dependence were included. In M2 the educational level of the dependent person was added, and in M3 the size of the municipality of residence and the regional unemployment rate (to capture regional differences in the labour market) were included. Sample weights were used in all estimates.

**Fig. 1:**
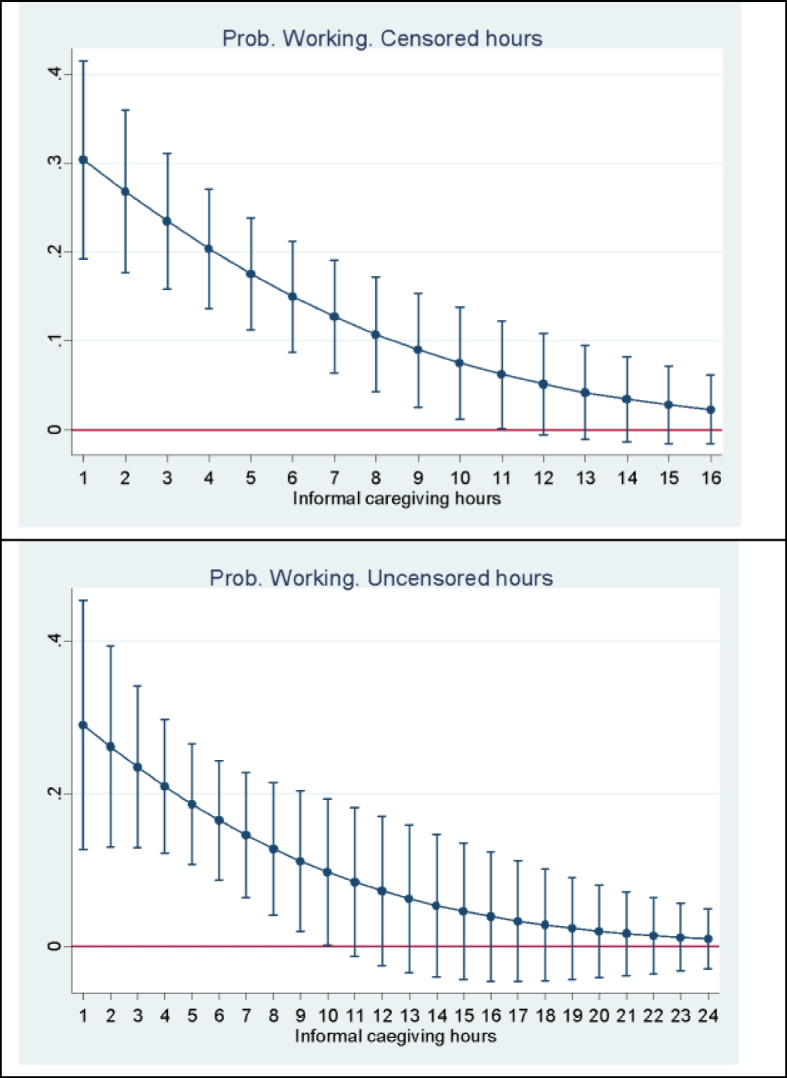
Predicted probabilities of being working conditioned on the number of (censored/uncensored) informal caregiving hours. Upper figure shows the predicted probability of being working after the estimation of a progit model for this outcome using as explanatory variables informal caregiving hours (censored at 16 h/day), sex, education and size of municipality of residence. Lower figure shows the predicted probability of being working after the estimation of a probit model for this outcome using as explanatory variables informal caregiving hours (maximum 24 h/day), sex, education and size of municipality of residence. Vertical bars denote confidence intervals at 95% significance

The results showed that the probability of being employed was 11.5 pp lower in men, but sex was not significant for the probability of being unemployed. Age exerted a negative and significant effect, and every 10 years of life, the probability of being employed decreased by 6 pp. Concerning the level of dependence: (i) the probability of being employed decreased by 18 pp for moderately dependent people and by 21 pp for highly dependent individuals; (ii) being severely dependent increased the probability of being unemployed by 71 pp (no highly dependent individuals looking for a job). Having a higher educational level increased the probability of being employed (by 16 pp). Also estimated was a probit model for the probability of being in work, replacing the degree of dependence by the number of informal caregiving hours (and maintaining the other explanatory variables). For a better interpretation of the results, Figure 1 in Appendix 2 shows the predicted probability of being in work conditioned by the number of informal caregiving hours. The results using censored or uncensored hours were consistent. When the person suffering from fibromyalgia started receiving one hour of informal care, the probability of being in work was very low (30%) and fell to 10% when receiving five hours of informal daily care.

Table 4 shows the number of hours of informal care provided. A total of 157,086 people out of the estimated population of 275,841 received informal care. The average number of hours per week amounted to 50.5 h (if one censors the maximum daily care time at 16 h) or 60.9 h (uncensored time). It can be seen that there was a large difference between people whose level of dependence was estimated to be high/severe and those whose level of dependence was moderate/non-eligible, according to the official scale applied in Spain to determine those levels: from 46.8 h/week for moderately dependent to 67.7 h/week for highly dependent, using censored hours, or between 54.2 h/week and 81.7 h/week using non-censored hours. There was a significant difference between the number of hours of care provided by male caregivers (58.5 h/week) and the number provided by female caregivers (40.8 h/week). So there was a difference in men´s favour of 17.6 h/week (censored hours) or of 25.5 h/week (uncensored hours).

**Table 4:** Informal caregiving hours

	Max. 16 h/day	Max. 24 h/day
**Informal caregivers (at population level)**	**157,086**	**157,086**
**Panel A: Weekly informal caregiving hours for average caregiver**		
Dependency degree: hours per week		
Non eligible	44.42	54.44
	(37.53)	(56.65)
Moderate dependent	46.82	54.15
	(34.74)	(49.75)
Severe dependent	63.63	79.11
	(37.36)	(58.77)
Highly dependent	67.67	81.67
	(36.31)	(56.81)
Average	50.49	60.89
	(37.32)	(55.69)
By informal caregiver’s sex		
Men	58.45	72.69
	(38.67)	(60.25)
Women	40.82	47.17
	(34.47)	(48.25)
Days per week	6.54	6.54
	(1.11)	(1.11)
**Panel B: Annual informal caregiving hours for caregiver**		
Non eligible	2,316	2,839
Moderate dependent	2,442	2,823
Severe dependent	3,318	4,125
Highly dependent	3,528	4,258
Average	2,527	3,051
**Panel C: Annual informal caregiving hours for total caregivers**		
Non eligible	127,623,059	156,409,339
Moderate dependent	84,805,262	98,070,628
Severe dependent	50,517,386	62,812,148
Highly dependent	40,105,102	48,402,709
Total	303,050,810	365,694,823
**Panel D: Distribution of annual informal caregiving hours (%)**		
Non eligible	42.11%	42.77%
Moderate dependent	27.98%	26.82%
Severe dependent	16.67%	17.18%
Highly dependent	13.23%	13.24%

Panel A shows daily informal caregiving hours

Panel B shows the annual number of informal caregiving

Panel C shows the yearly number of informal caregiving hours at population level

Panel D show the percentual distribution of yearly informal caregiving hours

Left part of the table considers that the maximum number of caregiving hours is 24. Right part of the table considers that the maximum number of caregiving hours is 16, so all caregivers who report a number of daily hours of care greater than 16 are censored at 16 h

All figures have been computed using population sampling weights

Source: own work using EDAD(2020)

These figures, extrapolated to annual terms, resulted in between 2,527 and 3,051 h per carer (with and without censoring of care time). When converted into population terms, the result was between 303 and 366 million hours of informal care provided (42.11% corresponding to non-eligible, 27–98% to moderately dependent, 16.67% to severely dependent and 13.23% to highly dependent).

Table 8 in Appendix 2 shows the regression analysis performed on the censored number of hours of informal care per week (estimates with the number of uncensored hours yield similar results and are available upon request). In all models the following were introduced as explanatory variables: care receiver characteristics (age, sex, education level, marital status), caregiver characteristics (age, sex, education level, kinship relationship with the caregiver), support received by the caregiver (cash subsidy, professional home care, daycare centre, household employee) and size of the municipality of residence. There may be an endogenous relationship between informal care hours and formal support [46]. The intention in including these variables was not to seek a causal relationship, but rather a correlation between them. The two models by which to estimate were: (i) with the degree of dependence of the care receiver (non-eligible being the omitted category) and (ii) with the description of the tasks performed by the caregiver (in which case the degree of dependence was not included). Additionally, both models estimated the total number of caregivers and differentiated according to the sex of the caregiver.

Gender of the dependent person was not a significant variable, but age (with a positive effect on caregiving hours) certainly was, although only for male caregivers. Caring for a moderately dependent person increased by 5.5 h/week of additional care, 13.9 h/week for a severely dependent person and 23.7 h/week for a highly dependent person. Contingent on the sex of the caregiver, when caring for a moderately or severely dependent person, the hourly intensity of male caregivers was respectively 1.35 times or 1.71 times higher than that of female caregivers. In contrast, for female caregivers, the intensity was 1.65 times higher than that of male caregivers when caring for a highly dependent person.

With respect to caregivers’ characteristics, the number of hours of care increased among caregivers with elementary education (the effect being 4 times higher among female caregivers), and with respect to kinship relationship, it increased among child caregivers and mother caregivers of the dependent person.

Receiving a cash subsidy was associated with an increase of 19.2 h/week (the effect being 3.5 times higher for female caregivers than for male caregivers). In contrast, having a household employee was associated with a reduction of 17.2 h/week of caregiving (the effect being 1.6 times higher for male caregivers).

On the right-hand side of Table 8 in Appendix 2 are shown the estimates when the tasks performed by the caregiver are entered instead of the degree of dependence. It is observed that having to help with eating and drinking was associated with an increase of 16.1 h/week, but with a significant difference between male and female caregivers (22.2 h/week for men and 14.6 h/week for women, i.e. 7.6 h/week more for men). Significant differences were also found in favour of male caregivers for the following tasks: "dressing/undressing" (2.3 h/week), "changing body postures, moving" (2.2 h/week), "shopping/preparing meals" (2.7 h/week).

Table 5 shows the monetary value of informal care time using the contingent valuation and the replacement cost scenarios. In the former, the value of time per carer ranged from 8,557 euros per year (WTP) to 17,799 euros per year (WTA), in both cases considering the censoring of the maximum daily care time at 16 h. When this censorship was removed, the values increased to 10,321 euros (WTP) and 21,470 euros (WTA). The valuation by the replacement cost method led to much higher figures. The range of estimates was between 40,380 and 48,707 euros. The table also compares these estimated figures with the average salary in Spain in 2021, the average retirement pension and the GDP per capita.

**Table 5:** Valuation of annual caregiving hours per informal caregivers

	**Censored hours (max. 16 h/day)**			**Not censored hours (max. 24 h/day)**		
	**Contingent valuation**		**Replacement**	**Contingent valuation**		**Replacement**
	**WTP**	**WTA**		**WTP**	**WTA**	
Non eligible	7,687	15,990	36,275	9,420	19,596	44,457
Moderate dependent	8,102	16,853	38,235	9,369	19,490	44,216
Severe dependent	11,010	22,902	51,956	13,689	28,475	64,601
Highly dependent	11,708	24,355	55,254	14,131	29,394	66,686
Average	8,557	17,799	40,380	10,321	21,470	48,707
**Average valuation with respect to average wage (2021)**						
Average	33%	69%	156%	40%	83%	188%
**Average valuation with respect to average retirement benefit (2021)**						
Average	60%	125%	283%	72%	150%	341%
**Percentage with respect to per capita GDP (2021)**						
Average	33%	69%	158%	40%	83%	191%

WTP: willingness to pay; WTA: willingness to accept

WTP (Garrido-García et al., 2015) and WTA (Oliva-Moreno et al., 2019)

GDP per capita (2021): 25,498 €

Average wage (2021): 25,896 €/year

Average retirement benefit (2021): 14,277 €/year

The lower part of Table 5 compares the estimate of the value of informal care with the average wage, the average retirement pension and the GDP per capita. Focusing on the estimates obtained with censored hours, informal care represented between 33 and 156% of the average wage, between 60 and 283% of the average retirement pension and between 33 and 158% of the GDP per capita.

Table 6 summarises the costs associated with the productivity losses of people with fibromyalgia and the value of informal care received, considering the different valuation scenarios shown, and differentiating whether the person with fibromyalgia is male or female. Thus the most conservative economic-impact scenarios were those that estimated the value of care time in a censored way and with the WTP estimated by Oliva et al. [35]. When the replacement cost technique was used, the estimated values were much higher. The range of values then oscillated between 2,443.6 and 7,164.8 million euros per annum (p.a.). In the first scenario, the estimated cost ranged between 2,443.6 and 2,651.4 million euros p.a. when censoring was applied on the maximum daily care time, and between 3,529.8 and 3,962.4 million euros p.a. when the maximum care time was not censored. In scenario 2, the values ranged from 6,183.8 to 7,164.8 million euros p.a.. An important result to highlight is that, in all the scenarios considered, the weight of the cost associated with fibromyalgia suffered by women was 90%, while the remaining 10% corresponded to men.

**Table 6:** Social costs (informal care + labour productivity losses) associated to fibromyalgia. Population level

	Men	Women	Total
Informal care valuation (censored hours)			
Estimation 1a WTP Oliva. 16 horas	100.9	904.7	1,005.6
Estimation 1b WTA Garrido. 16 horas	117.1	1,096.3	1,213.4
Estimation 2. Replacement rate. 16 horas	476.4	4,269.4	4,745.8
Informal care valuation (uncensored hours)			
Estimation 1a bis WTP Oliva. 24 horas	210	1,881.8	2,091.8
Estimation 1b bis.WTA Garrido. 24 horas	243.6	2,280.8	2,524.4
Estimation 2bis Replacement rate. 24 horas	552.7	5,174.1	5,726.8
Labour costs			
Reduced labor participation (patient)			
Average wage (by sex)	132.0	1,306.0	1,438.0
Minimum wage	62.5	738.6	801.1
Total (base case)			
Estimation 1a	232.9	2,210.7	2,443.6
Estimation 1b	249.1	2,402.3	2,651.4
Estimation 2	608.4	5,575.4	6,183.8
Estimation 1a bis	342.0	3,187.8	3,529.8
Estimation 1b bis	375.6	3,586.8	3,962.4
Estimation 2 bis	684.7	6,480.1	7,164.8
Total (conservative estimate)			
Estimation 1a	163.4	1,643.3	1,806.7
Estimation 1b	179.6	1,834.9	2,014.5
Estimation 2	538.9	5,008.0	5,546.9
Estimation 1a bis	272.5	2,620.4	2,892.9
Estimation 1b bis	306.1	3,019.4	3,325.5
Estimation 2 bis	615.2	5,912.7	6,527.9

Source: own elaboration from EDAD 2020

Unit: million euros. Year: 2021

Base case: using average wages, adjusted by sex; conservative estimate: using minimum wages

When the weight of the two main cost items was valued (informal care time and loss of labour productivity), the result strongly depended on the method used to value informal care time. Thus, when the chosen method was replacement cost, the weight of the value of informal care as a proportion of the total cost ranged from 75.7% (censoring the maximum daily care time) to 79% (uncensored), the remaining 24.3% and 21% being the value of the labour productivity losses of fibromyalgia sufferers. When the contingent valuation scenarios were considered, the weight of informal care represented between 39.8% and 44.3% of the total cost when the maximum daily care time was censored. In contrast, when censoring was removed, these weights represented between 57.9% and 62.4% of the total cost.

## Discussion and Conclusions

The results of our analysis reveal a high socio-economic cost borne by fibromyalgia sufferers and their affective environment. If we take into account the estimates obtained in scenarios 1a and 2 (scenarios with daily hours of informal care censored), the range of the estimate is between 2,443.6 and 6,183.8 million euros p.a., with 2021 being the reference year for the valuation. Given the population size provided by EDAD 2020, this leads to an annual cost per person of between 8,859 euros p.a. (estimate 1a) and 22,418 euros p.a. (estimate 2). If the most conservative contingent valuation scenario is used, the weight of labour productivity losses is 58.9%, with informal care accounting for 41.1%, while if replacement cost is used, the weight of labour productivity losses is reduced to 20.0%, with informal care accounting for the remaining 80.0% of the cost. Both the total social cost figure and its distribution between productivity losses and informal care are strongly conditioned by the method used for the valuation of informal care time. However, regardless of the valuation methods used, the cost figures obtained are very high, both at the population level and in terms of cost per patient. One aspect on which epidemiological and economic studies agree is that the majority of patients suffering from fibromyalgia are women. The proportion usually found in the literature is 90% or higher, which is congruent with the percentage in our study. Considering the cost figures, 90% of the costs are also due to fibromyalgia suffered by women.

In contrast to other diseases, we have not identified any studies in the literature that estimate the social cost of fibromyalgia on a national scale. The difficulty in diagnosing this disease results in considerable uncertainty about the difference between the diagnosed prevalence and the real prevalence of the disease. This may be one reason why it is difficult to combine epidemiological and economic information to extrapolate results from other studies to a national estimate. In our work we had the advantage of having a nationwide survey that allowed us to identify 699 people suffering from fibromyalgia and some type of disability, and the methodological support of the National Institute of Statistics, which provided the weights to transfer these figures to the population scale. We believe that this is a strength of the work and that our results can serve as a future basis for comparison for work carried out in other countries. To put into context the relevance of the total estimated figures at national level, and taking into account the values of the estimates obtained in scenarios 1a and 2 (2,443.6 and 6,183.8 million euros, respectively), the figures would represent between 2.58% and 6.63% of public health expenditure in the same year (which accounted for 7.7% of GDP in 2021) or between 25.19% and 63.75% of the total cost of the System for Autonomy and Care for Dependence (which accounted for 0.9% of GDP).

The results of our work should serve to raise the visibility of a disease that places an enormous social and economic burden on sufferers and on society as a whole. From the perspective of the organisation of healthcare resources, shortening the diagnostic times for this disease would help to reduce the anxiety caused to patients by delays in diagnosis. From a treatment perspective, unfortunately, in their systematic review Mascarenhas et al. [18] found that most of the identified treatments for fibromyalgia treatments were ineffective. Only cognitive behavioural therapy for pain, as well as the use of antidepressant medication and central nervous system depressants can be effective in reducing pain and improving quality of life, although the effects found were limited. Other recent work proposing multi-component interventions seems to offer an efficient result [47]. Nevertheless, current evidence is lacking for most therapies [18], and further research is needed to develop better treatment options [48].

The results should invite public decision-makers to reflect on the occupational fragility of people affected by fibromyalgia. Our study adds to previous work that has identified the severe impact on employment of people with fibromyalgia. These studies identify both the reduction in the employment rate of people with fibromyalgia from the time of diagnosis or even earlier (development of symptoms before receiving a clinical diagnosis) [17, 49–51], and the low labour participation of these people as the disease progresses [52–56]. Mannerkorpi and Gard [57] state that the need to take breaks and the deterioration of physical capacity are the main obstacles to maintaining their work activity. In this regard, Sallinen et al. [58] have described how adaptations of work schedules and tasks can enable people with fibromyalgia to continue working. Briones-Vozmediano et al. [59] revealed the lack of information and awareness about the disease among supervisors and managers, which could lead them not to confess their medical condition for fear of being fired. Also, they emphasized that personal relationships with coworkers might be clouded, due to their lack of understanding of the patients' reduced performance, while absences, mistakes made or forgetfulness might lead to later conflicts with employers. Changes in employment status cause a high percentage of households reporting difficulties in making ends meet and experience an increase in expenses associated with illness [15]. Laroche et al. [60] report a rise in feelings of perceived unfairness among unemployed fibromyalgia patients and in lower income households.

This results in a situation of income loss and vulnerability that is aggravated by three factors. First, the number of respondents in the EDAD 2020 whose work activity was modified as a result of fibromyalgia, but who were still employed, was extraordinarily low. This contrasts sharply with the low employment rates observed in this population. A longitudinal survey, or at least one that includes retrospective questions, would be needed to discover the respondents' work history and how it was affected by the onset of symptoms accompanying fibromyalgia, and also how long it took from the onset of symptoms to the time of diagnosis and whether they left employment voluntarily or were dismissed. Secondly, in several countries people with fibromyalgia have been found to have difficulty in obtaining a pension for permanent incapacity for work. For instance, in Spanish legislation, the granting of a pension of this kind requires not only the presence of a serious health impairment but also the permanent and definitive nature of the lesions and the fact that they have a decisive influence on the person's ability to work. Given the nature of this symptomatic disease, its unknown aetiology and the difficulty of proving the permanence of the condition, it is not unusual for medical courts to refuse to grant permanent disability status and for affected persons to have to take their cases to court in proceedings that can last for many years [61]. Along the same lines, the recent debate in the French Senate about the consideration that fibromyalgia should have,^3^ which has resulted in the registration in the Assemblée Nationale of a Proposed Law for the recognition of fibromyalgia as a long-term condition, is very significant.^4^ The third aspect that we would like to highlight is the fact that the low employment rates observed in people with fibromyalgia will in the future result in contributory or non-contributory pensions of amounts well below the average contributory pensions. Several studies have found that the presence of a favourable psychosocial work environment may be important for the maintenance of work capabilities [49], but, certainly, more research is needed on specific interventions in the work environment [13, 15, 17]. It would also appear that regulatory changes to provide people with fibromyalgia with social protection on a parity with people with other recognised health conditions is an urgent matter which should be addressed even in countries with well-established welfare systems.

A limitation on our work is the lack of data about the use of healthcare resources by people with fibromyalgia. However, the literature seems to agree that in patients with fibromyalgia, social costs outweigh healthcare costs. Thus, the values obtained by Winkelman et al. [22] for the labour losses of 299 fibromyalgia patients residing in France and Germany amounted to 6,990 and 5,491 euros respectively, while healthcare costs per patient were estimated at 910 and 1,765 euros (base year, 2008). Also in France, Perrot et al. [23] for a sample of 88 patients estimated an annual health care cost of 808 euros, plus 103 euros of non-health professional care and 6,990 euros of indirect costs (year of estimation: 2008). Although differences in means by severity levels were identified, these differences were not statistically significant. In the work of Chandran et al. [29] the social costs (productivity losses and informal care) of 203 patients in the United States amounted to $5,366, $20,556, and $33,139 per patient with mild, moderate, and severe fibromyalgia respectively (base year 2009). The weight of social costs in total costs (including healthcare costs) ranged from 52.5% to 78.4%, depending on the degree of severity of the fibromyalgia. In the paper by Garihpoor et al. [24] relating to 62 Iranian patients, social costs are estimated at $2947 (year of assessment: 2017) for a 6-month estimation period, with non-healthcare costs outweighing healthcare costs (51% vs. 49%). Boonen et al. [31], studying 70 patients living in the Netherlands with fibromyalgia, estimated an annual cost of 7,814 euros (year of estimate 2002), of which 16.8% were healthcare costs, 50.3% were direct non-healthcare costs (professional and informal care) and 32.9% were labour losses. Verboot et al. [30], using data from 280 patients living in the Netherlands, estimated a healthcare cost of 2,944 euros and labour productivity losses of 5,731 euros (year of assessment: 2012). In Spain, the most recent work carried out [28], using information from 232 patients, estimates a monthly healthcare cost of 423 euros and labour losses of 742 euros (annualised to 5,076 and 8,904 euros respectively) (year of estimate 2010). A summary table (see Table 9) of these results can be found in Appendix 2.

From the above results, it also appears that the estimated social costs per patient in our work are among the highest in the literature reviewed, with the exception of the work of Chandran et al. [29] for the USA. There are several reasons for this. First, the items in which social costs are included differ in the reviewed papers. Thus, in our paper, labour productivity losses and the value of informal care time were included. The use of professional care services was not included, although their use reported in the EDAD 2020 by people with fibromyalgia was very low.. A second factor that helps to explain these differences is the valuation methods used. In our own research, the choice of valuation method for informal care strongly influences the final estimate. In this sense, there is no international consensus about which methods are the best to estimate the value of productivity losses or to apply to informal care time [36, 37]. The choice between the friction cost or human capital method, in the case of labour productivity losses, or between the replacement cost, opportunity cost or contingent valuation method (the latter being the most common in the case of informal care) can lead to big differences in the monetary valuation of the resources used or lost. Thirdly, the composition of the groups of patients analysed in the studies may lead to relevant differences that need to be considered in the comparison of the results. In our study, participants are selected to be surveyed in depth if in a previous round they state that they have some kind of disability in vision, hearing, communication, learning, mobility, self-care, domestic life or interpersonal relationships. Although the presence of a disability does not necessarily mean a limitation in carrying out basic or instrumental activities of daily living, 57% of people with fibromyalgia stated that they require personal care to carry out their usual activities. An aspect that remains open for future research is an estimation of labour losses adjusted for gender, age, educational attainment and work experience. However, this would require a longitudinal study design combining a prospective (from the time of diagnosis) and retrospective approach (as the diagnosis may be made long after the onset of the first symptoms of the disease). Such a design would help to correct for biases and achieve a better estimate of labour losses in terms of employment and wages. Such a design would help to correct for biases and achieve a better estimate of labour losses in terms of employment and wages.

Finally, we would not want to conclude this section without referring to the importance of non-professional or informal care. The contingent valuation scenarios show us a conservative scenario of the value of informal care time, while the scenarios in which replacement cost valuation is applied inform us of the resources, public and private, that would have to be mobilised in the event of having to replace previous care by professional care. In both cases, even in the conservative ones, the importance of informal care is shown. In this regard, the literature analysing not only the value of this type of care, but also other consequences of providing it, has grown significantly. There is now strong evidence that when caregiving is very intensive in terms of hours per week, when it lasts for a long time (years), or when it does not have adequate institutional or social support, it becomes a significant burden for caregivers, affecting their health, employment opportunities and socio-family relationships [62–67]. However, in the field of fibromyalgia, the existing literature about the effects of caregiving is very limited, as is the evaluation of interventions aimed at improving caregivers' information, skills and quality of life [68, 69]. In the same sense, there is a need for further studies on the contingent valuation of care specifically provided to people with fibromyalgia. Contingent valuation of the time of informal care provided to people with fibromyalgia would help to establish more adjusted values, as well as open up other studies that analyse the relationship between such valuation and care times, psychological burden, perceived social support and types of care provided.

Consequently, and as a final conclusion, we can stress that more research is needed to reveal the economic and social burden on people suffering from fibromyalgia and on their affective environment, as well as to identify those healthcare, regulatory and social interventions that improve the well-being of patients and caregivers.

## Data Availability

The data used in the analysis are in the public domain. Any researcher can access them through the National Institute of Statistics at the link: https://www.ine.es/dyngs/INEbase/es/operacion.htm?c=Estadistica_C&cid=1254736176782&menu=resultados&idp=1254735573175

## Appendix 1

### The Spanish dependence system

The present Spanish dependence system was approved by Law 39/2006, of December 14, 2006, on the Promotion of Personal Autonomy and Care for Persons in a Situation of Dependence (SAAD). This law was a major reform since it universalized access (not financing) and benefits (financial and in kind) for dependent people. Prior to the SAAD, subsidies were granted by local administrations on a means-tested basis, and were financed from the limited budgets of local administrations [70].

To determine whether a person is entitled to receive any of the SAAD benefits, an assessment of his or her needs for support in personal and instrumental activities of daily living is carried out. Royal Decree 504/2007, of April 20, 2007, approved the dependence scale established by Law 39/2006, of December 14, 2006, on the Promotion of Personal Autonomy and Care for Persons in a Situation of Dependence.

Once a certain level of dependence has been recognized, an "individual care plan" is drawn up, listing the benefits that can be received, taking into consideration the preferences of the beneficiary and his or her family. Service benefits include services for the prevention of dependence and for the promotion of personal autonomy, telecare, home care, day/night centre and residential care. The autonomous communities (regions) are responsible for the accreditation and supervision of professional services.

Cash benefits can be of the following types: (1) a service-linked financial benefit, which is only granted when care through a public care service is not possible, (2) personal assistance allowances to facilitate the beneficiary's access to education and employment, and (3) financial subsidies for care in the family environment (to reward informal caregivers). These benefits are only granted when there is no corresponding benefit in kind. Cash benefits are incompatible with benefits in kind, except for telecare.

Since its inception, the SAAD has faced serious problems of uncertainty in both organization and funding, political differences between the government of the Central Administration and those of the Autonomous Communities (regions), as well as lack of recognition of the work of informal caregivers [71, 72].

The economic crisis (Great Recession) was coupled with the existing underfunding from its origins. In 2012, severe cutbacks were approved both in the amount of financial benefits and in the number of hours of home care. In addition, in 2019 and 2020, the general state budget bill was not approved, which meant that the 2018 budget was maintained. Finally, in 2022, an increase in the dependence budget of €2,902 million was approved, double the amount in 2018 [73].

## Appendix 2

**Table 7:** Probability that a patient with fibromyalgia is working and probability of being unemployed (marginal effects after probit)

	Prob (working)			Prob(unemployed)		
	M1	M2	M3	M1	M2	M3
Dependent’s characteristics						
Man	-0.115**	-0.118**	-0.105**	0.153	0.246	0.267
	(0.054)	(0.053)	(0.073)	(0.202)	(0.209)	(0.219)
Age	-0.007***	-0.008**	-0.006**	0.002	0.001	0.001
	(0.003)	(0.003)	(0.003)	(0.006)	(0.006)	(0.006)
Dependency degree						
Moderate dependent	-0.052	-0.056	-0.056	-0.037	-0.048	-0.003
	(0.052)	(0.051)	(0.051)	(0.118)	(0.119)	(0.121)
Severe dependent	-0.184**	-0.187***	-0.182***	0.658***	0.632***	0.716***
	(0.112)	(0.082)	(0.082)	(0.302)	(0.304)	(0.307)
Highly dependent	-0.214**	-0.219***	-0.212***			
	(0.102)	(0.101)	(0.101)			
Level of education						
Elementary		-0.077	-0.075		0.267	0.261
		(0.074)	(0.074)		(0.210)	(0.214)
High school		0.011	0.013		0.161	0.145
		(0.062)	(0.062)		(0.167)	(0.168)
College		0.173**	0.165**		-0.012	0.005
		(0.072)	(0.072)		(0.179)	(0.182)
Size of municipality						
50,000–100,000 inhabitants			0.077			-0.031
			(0.065)			(0.125)
20,000–50,000 inhabitants			0.086			-0.261**
			(0.059)			(0.129)
10,000–20,000 inhabitants			0.000			-0.082
			(0.063)			(0.151)
Less than 10,000 inhabitants			-0.032			0.018
			(0.055)			(0.137)
Constant	0.685***	0.566***	0.534***	0.222	0.162	0.215
	(0.143)	(0.157)	(0.160)	(0.306)	(0.356)	(0.382)
N	394	394	394	122	122	122
R2	0.350	0.386	0.397	0.239	0.276	0.313
F	24.079	24.537	23.412	21.173	21.345	21.280
P	0.001	0.000	0.000	0.001	0.000	0.000

Omitted variables: dependent characteristics (not eligible; has not completed elementary education), provincial capitals and municipalities with more than 100,000 inhabitants. M3 models include regional unemployment rate. Estimates obtained using sampling weights. The probability of being employed is calculated over the entire working age population. The probability of being unemployed is calculated over the active population

*Statistically significant (*p*<0.1)

**Statistically significant (*p*<0.05)

***Statistically significant (*p*<0.01)

**Table 8:** Regressions for the number of weekly informal caregiving hours. Maximum 16 h/week. Nonparametric robust regression estimates)

	Total	Caregiver’s sex		Total	Caregiver’s sex	
		Men	Women		Men	Women
**Dependent characteristics**						
Man	-3.43	-8.52	-1.64	-7.60	-25.43	-9.61
	(7.56)	(21.59)	(9.93)	(7.84)	(23.58)	(11.03)
Age	0.50**	0.71***	0.08	0.48**	0.82***	0.25
	(0.21)	(0.28)	(0.28)	(0.22)	(0.29)	(0.30)
Dependency degree						
Moderate dependent	5.45**	7.99**	5.91**			
	(2.09)	(3.04)	(2.64)			
Severe dependent	13.93***	19.20***	11.24***			
	(6.95)	(7.15)	(5.46)			
Highly dependent	23.71***	22.35***	36.86***			
	(8.15)	(10.15)	(11.49)			
Level of education						
Elementary	-6.78	-4.27	-10.80	-5.22	4.49	-13.06
	(6.40)	(11.50)	(7.79)	(6.70)	(12.31)	(8.45)
High school	-1.52	-4.34	1.10	-2.61	2.09	-2.98
	(6.58)	(11.75)	(8.76)	(6.64)	(12.30)	(9.29)
College	-1.19	12.10	-4.80	-5.13	17.66	-15.47
	(9.01)	(14.88)	(12.09)	(8.94)	(15.03)	(12.35)
Marital status						
Single	8.24	32.01*	-0.57	6.48	22.70	-1.93
	(10.38)	(17.33)	(14.18)	(10.72)	(18.39)	(14.61)
Married/cohabiting	4.05	20.67	0.12	1.81	14.14	-0.16
	(7.73)	(14.95)	(8.96)	(7.87)	(15.20)	(9.56)
Widow	-5.05	0.20	0.39	-5.47	-8.41	-1.88
	(8.29)	(18.49)	(9.07)	(8.25)	(18.76)	(9.20)
**Caregiver’s characteristics**						
Man	8.03	-	-	8.00	-	-
	(5.49)	-	-	(5.56)	-	-
Age	0.05**	0.27	0.04*	0.04	0.25	0.03
	(0.02)	(0.37)	(0.02)	(0.02)	(0.38)	(0.02)
Level of education						
Elementary	32.58***	14.33	48.46***	30.23***	11.18	47.11***
	(7.14)	(10.86)	(9.83)	(7.29)	(11.14)	(10.88)
High school	7.27	1.02	3.99	7.46	-2.80	10.74
	(5.89)	(9.36)	(7.86)	(5.91)	(9.37)	(8.00)
College	9.28	-4.06	8.73	6.31	-15.42	18.65*
	(7.30)	(12.59)	(9.95)	(7.36)	(12.93)	(9.60)
Kinship caregiver with respect to care receiver						
Spouse/partner	8.99	2.28	3.36	7.66	1.94	11.54
	(7.44)	(15.54)	(9.49)	(7.55)	(16.04)	(10.31)
Mother/father	19.69*	15.49	19.53***	22.48**	17.73	27.45***
	(10.38)	(17.04)	(8.47)	(10.41)	(17.54)	(11.08)
Daughter/son	12.36**	21.37***	-3.49	12.01**	17.10***	-4.56
	(5.21)	(7.80)	(7.43)	(5.28)	(6.97)	(7.97)
**Dependent receives:**						
Cash subsidy	19.16***	9.68***	35.08**	19.40***	9.65***	35.60**
	(9.63)	(3.47)	(14.60)	(9.83)	(3.10)	(10.20)
Professional home care	-8.57	11.35	-15.96*	-7.47	11.69	-14.73*
	(7.07)	(14.47)	(8.35)	(7.19)	(14.29)	(8.67)
Daycare center	-2.26	13.98	-17.27	-2.47	13.76	-17.53
	(14.30)	(23.83)	(19.25)	(14.76)	(25.02)	(19.84)
Telecare	7.47	5.94	3.51	7.66	5.60	3.50
	(7.56)	(13.62)	(9.12)	(7.57)	(14.11)	(9.42)
Help from household employee	-17.16**	-22.14**	-13.99**	-17.28**	-21.82**	-14.08***
	(7.68)	(10.37)	(6.50)	(7.68)	(10.52)	(6.52)
**Size of municipality**						
50,000–100,000 inhabitants	-10.86	-21.13*	-3.39	-9.83	-21.06	-1.97
	(7.36)	(12.63)	(9.28)	(7.39)	(12.70)	(9.45)
20,000–50,000 inhabitants	-6.97	-4.41	-13.66	-4.72	-0.75	-10.06
	(6.97)	(9.63)	(11.16)	(7.04)	(9.64)	(11.44)
10,000–20,000 inhabitants	-5.47	-2.27	-15.02	-4.98	-0.50	-14.95
	(6.88)	(11.15)	(9.07)	(6.92)	(10.99)	(9.30)
Less than 10,000 inhabitants	-7.34	-16.60	-3.10	-6.04	-11.63	-2.17
	(6.21)	(10.29)	(8.17)	(6.22)	(10.33)	(8.27)
Task primarily undertaken by informal caregiver						
Eating, drinking, feeding				16.07**	22.15**	14.56**
				(8.34)	(10.55)	(7.14)
Dressing / undressing / fastening of shoes				13.77**	16.08**	13.75**
				(5.05)	(6.91)	(6.84)
Grooming / dressing				3.81	2.09	4.25
				(5.14)	(9.09)	(6.87)
Going to the toilet, changing diapers, etc.,				3.25	3.15	3.23
				(7.63)	(11.95)	(10.20)
Changing posture/moving/keeping body in a certain posture				13.74**	14.97**	12.77**
				(5.77)	(6.79)	(5.82)
Walking or moving around the house				2.73	2.08	2.79
				(6.03)	(8.46)	(9.48)
Going outside, going up or down stairs				1.55	1.46	4.07
				(4.56)	(7.22)	(6.30)
Taking medication, going to doctor's appointments				5.17	4.66	6.87
				(4.64)	(7.30)	(6.19)
Shopping, preparing meals				13.91**	15.39**	12.71**
				(6.76)	(6.24)	(6.74)
Carrying out other household chores				6.55	6.97	6.74
				(5.33)	(8.22)	(7.58)
Using the telephone, computer				4.28	4.86	5.33
				(12.11)	(20.08)	(16.57)
Constant	-0.25	-14.62	26.13	-63.62*	-46.81	-103.70**
	(17.84)	(29.18)	(23.40)	(34.34)	(54.77)	(50.70)
N	398	122	276	283	161	122
R2	0.32	0.36	0.45	0.35	0.43	0.50
F	13.66	12.12	12.76	13.19	12.98	12.37
p	0.00	0.00	0.00	0.00	0.01	0.00

Omitted variables: caregiver characteristics (has not completed elementary education; separated/divorced), dependent characteristics (not eligible; has not completed elementary education; separated/divorced), provincial capitals and municipalities with more than 100,000 inhabitants. Estimates obtained using sampling weights. Robust estimates

*Statistically significant (*p*<0.1)

**Statistically significant (*p*<0.05)

***Statistically significant (*p*<0.01)

**Table 9:** Results of cost of fibromyalgia studies conducted from a societal perspective

						Annualized Costs per patient		
Author	Country	Sample size	Base year	Monetary unit	Severity	Health care costs	non health care costs (including informal care)	Labour losses
Winkelman et al. [22]	France & Germany	299	2008	€				
	France				mild	564	93	4,816
					moderate	949	108	5,576
					severe	794	103	9,190
	Germany				mild	1133	140	786
					moderate	989	295	5,004
					severe	1,996	284	8,466
Perrot et al. [23]	France	88	2008	€	Total	808	103	6,990
					mild	564	93	4,816
					moderate	949	108	5,576
					severe	794	103	9,190
Garihpoor et al. [24]	Iran	62	2017	$US	Total	5,634	2,996	2,898
					mild	6,254	3,414	6,264
					moderate	6,022	1,788	1,664
					severe	4,756	3,674	2,896
Rivera et al. [28]	Spain	232	2010	€	Total	5,905		8,909
Chandran et al. [29]	USA	203	2009	$US	mild	4,852	936	4428
					moderate	5,660	5,892	14,664
					severe	9,316	9,144	23,996
Verboot et al. [30]	Netherlands	280	2012	€	Total	2,016	2,347	4,440
Boonen et al. [31]	Netherlands	69	2002	€	Total	1311	3,930	2,573

Source: own elaboration based on the information contained in the above-mentioned articles

## Footnotes

1 Source: INE, Metodología https://www.ine.es/metodologia/t15/meto_edad_2020.pdf.

2 Source: Servicios Sociales para Personas Mayores en España 2021; https://imserso.es/el-imserso/documentacion/estadisticas/servicios-sociales-dirigidos-a-personas-mayores-en-espana-diciembre-2021.

3 https://www.senat.fr/questions/base/2022/qSEQ221204417.html; https://www.senat.fr/questions/base/2023/qSEQ230607230.html#:~:text=L'organisation%20mondiale%20de%20la,la%20classification%20internationale%20des%20maladies

4 https://www.assemblee-nationale.fr/dyn/16/textes/l16b1218_proposition-loi
